# Two Distinct Modes of Lysis Regulation in *Campylobacter Fletchervirus* and *Firehammervirus* Phages

**DOI:** 10.3390/v12111247

**Published:** 2020-10-31

**Authors:** Athina Zampara, Stephen J. Ahern, Yves Briers, Lone Brøndsted, Martine Camilla Holst Sørensen

**Affiliations:** 1 Department of Veterinary and Animal Sciences, University of Copenhagen, Stigbøjlen 4, 1870 Frederiksberg C, Denmark; athina@sund.ku.dk (A.Z.); s.j.ahern86@gmail.com (S.J.A.); lobr@sund.ku.dk (L.B.); 2 Department of Biotechnology, Ghent University, Valentin Vaerwijckweg 1, 9000 Gent, Belgium; yves.briers@ugent.be

**Keywords:** *Campylobacter* phages, lysis regulation, endolysins, lysis inhibitor

## Abstract

*Campylobacter* phages are divided into two genera; *Fletchervirus* and *Firehammervirus*, showing only limited intergenus homology. Here, we aim to identify the lytic genes of both genera using two representative phages (F352 and F379) from our collection. We performed a detailed in silico analysis searching for conserved protein domains and found that the predicted lytic genes are not organized into lysis cassettes but are conserved within each genus. To verify the function of selected lytic genes, the proteins were expressed in *E. coli*, followed by lytic assays. Our results show that *Fletchervirus* phages encode a typical signal peptide (SP) endolysin dependent on the Sec-pathway for translocation and a holin for activation. In contrast, *Firehammervirus* phages encode a novel endolysin that does not belong to currently described endolysin groups. This endolysin also uses the Sec-pathway for translocation but induces lysis of *E. coli* after overexpression. Interestingly, co-expression of this endolysin with an overlapping gene delayed and limited cell lysis, suggesting that this gene functions as a lysis inhibitor. These results indicate that *Firehammervirus* phages regulate lysis timing by a yet undescribed mechanism. In conclusion, we found that the two *Campylobacter* phage genera control lysis by two distinct mechanisms.

## 1. Introduction

Bacteriophages have evolved diverse lysis mechanisms to liberate progeny phages from infected host cells. Phage-induced lysis of bacterial cells relies on the osmotic imbalance caused by degradation of the cell wall after a coordinated action of bacteriophage-encoded lytic proteins. The main lytic proteins belong to three functional classes: endolysins, holins and spanins, that often are encoded by genes organized into a gene cluster, known as a lysis cassette [1,2].

Endolysins are enzymes produced in the cell cytosol that degrade the peptidoglycan layer by targeting different types of peptidoglycan bonds. Endolysins are highly diverse with various catalytic activities and architectures but can often be predicted due to conserved catalytic domains [3,4]. In order for endolysins to target peptidoglycan, access to the periplasmic space is a prerequisite. Translocation across the cytoplasmic membrane and activation of endolysins in the periplasm are some of the mechanisms that phages utilize to control the timing of lysis [5,6]. In phage lambda, holins ensure that endolysins gain access to the periplasm [7]. Holins are transmembrane proteins that accumulate harmlessly in the cytoplasm until triggering the formation of holes in the cytoplasmic membrane, through which the enzymatically active endolysins are exported. Holins are highly diverse with more than fifty gene families described and are further categorized into three classes based on the number of transmembrane domains (TMDs) and topology [6,8]. Holin inhibitors, known as antiholins, can delay the host cell lysis timing by blocking holin multimerization and thereby hole formation [9]. However, multiple endolysins have been identified that escape the cytoplasm independent of a holin. They rely on the Sec-pathway and here the holins function as endolysin activators [10,11,12,13].

The Sec-pathway is a universal bacterial export system that catalyzes the translocation of proteins across the cytoplasmic membrane. The Sec-pathway targets endolysins containing signal peptides (SPs), such as *Oenococcocus oeni* phage fOg44 endolysin Lys44 [10,11] and endolysins with signal-arrest-release (SAR) domains as found in the N-terminal domain of phage P1 endolysin [12]. In both cases, holins function as lysis timing regulators by causing membrane depolarization that activates endolysins after translocation. SP-endolysins possess a clear N-terminal signal sequence divided into three regions (N, H and C) and are situated distantly from the catalytic domain. The H-region of the signal sequence is composed of strongly hydrophobic amino acids, which is cleaved off after translocation to the periplasm [14]. In contrast, SAR domains contain an H-region that is composed of only weakly hydrophobic residues and they lack the C-region. Therefore, they are not cleaved after export to the periplasm, but rather remain tethered to the inner membrane in an inactive form [12,15,16]. Activation of SAR endolysins occurs by conformational changes that take place upon release from the inner membrane. The release is caused by pinholins that depolarize the membrane, resulting in loss of the interaction of the positively charged N-region of the SAR with the negatively charged membrane, allowing refolding and therefore activation of SAR endolysins. Recently, other proteins have been shown to target endolysins to the Sec-pathway, functioning similar to chaperones, leading to cell lysis in the absence of a holin, as has been described for *Mycobacterium smegmatis* phage Ms6 [13].

In addition to peptidoglycan degradation, for most phages of Gram-negative hosts, the disruption of the outer membrane is required for successful cell lysis. Spanins are responsible for this process, often comprising an inner membrane protein (i-spanin) and an outer membrane lipoprotein (o-spanin) that form complexes spanning the periplasm [17]. Recent work suggests that spanins destroy the outer membrane by fusing the inner and outer membrane after degradation of the peptidoglycan by the endolysins [18,19,20,21].

Phages infecting *Campylobacter* possess unique genomic features and belong to two genera; *Fletchervirus* and *Firehammervirus*, both of which are highly unrelated to other phage genera [22,23]. While phages within a genus are highly homologous, phages of the two different genera recognize different receptors and display only very limited homology [24,25]. To date, thirteen *Campylobacter* phages have been sequenced, with nine belonging to *Fletchervirus* [23,26,27,28,29,30] and four belonging to *Firehammervirus* [31,32,33]. Previously, only few gene functions have been predicted and lytic genes have not been fully characterized. Spanins have not been identified in either genus, while putative holins and endolysins have been predicted, but without experimental confirmation [30,32]. Here, we aim to identify and characterize the lytic genes of both *Campylobacter* phage genera using phages F352 and F379 as representatives of *Fletchervirus* and *Firehammervirus*, respectively. Our results show that *Fletchervirus* phages encode a typical SP-endolysin dependent on the Sec-pathway for translocation and on a holin for activation. Similarly, *Firehammervirus* phages also encode an endolysin that is dependent on the Sec-pathway for translocation. However, these phages appear to possess a novel lysis inhibitor located upstream and overlapping this endolysin. Thus, a novel mechanism for regulating cell lysis is employed by phages belonging to *Firehammervirus* genus, further contributing to our understanding of the biology of these unique phages.

## 2. Materials and Methods

### 2.1. Bioinformatic Analysis and Annotation of Lytic Genes

Phage F352 (Genbank accession No. MT863717) belonging to *Fletchervirus* and *Firehammervirus* phage F379 (Genbank accession No. MT932329) (Table S1) were analyzed for the presence of lytic genes using InterProScan [34], TMHMM [35], TOPCONS [36], BLASTP [37], HMMER [38], SignalP [39] and HHpred [40]. Spanin genes were identified using CPT FindSpan on the CPT Galaxy instance.

### 2.2. Construction of Protein Expression Plasmids

Genes predicted to be involved in the phage lysis were synthetically made based on the DNA sequences of the lytic genes from representative phages F352 and F379 and inserted into pET-28a(+) using GeneCust (Dudelange, Luxembourg) service (Table S1). All constructs were further confirmed by sequencing (GeneCust).

#### 2.2.1. Construction of pAZ01

A 570 bp fragment including *lys (F352_101)* and an additional TAA stop codon was synthetically produced and cloned into pET28a(+) by using the NdeI and XhoI restriction sites, producing the endolysin from phage F352 with an N-terminal his-tag.

#### 2.2.2. Construction of pAZ02

A 285 bp fragment including *hol (F352_033)* and an additional TAA stop codon was synthetically produced and cloned into pET28a(+) by using the NdeI and XhoI restriction sites, producing the holin from phage F352 with an N-terminal his-tag.

#### 2.2.3. Construction of pAZ03

A 895 bp fragment including *lys (F352_101)* and downstream *hol (F352_033)* was synthetically produced and cloned into pET28a(+) by using the NdeI and XhoI restriction sites. An additional TAA stop codon was added in both genes to ensure translation termination. The two genes were connected by a 40 bp nucleotide sequence (TCTGTGTGATTTATACATGGTGTGAGCTGGAAGGAGATAA), found upstream of *hol (F352_033*) but with slight modifications (underlined) in order for both genes to possess similar ribosomal binding sites. Expression of these two genes produced the endolysin with an N-terminal his-tag and the holin from phage F352.

#### 2.2.4. Construction of pAZ04

A 678 bp fragment including *lys (F379_025)* and an additional TAA stop codon was synthetically produced and cloned into pET28a(+) by using the NdeI and XhoI restriction sites, producing the endolysin from phage F379 with an N-terminal his-tag.

#### 2.2.5. Construction of pAZ05

A 327 bp fragment including *F379_024* plus an additional TAA stop codon was synthetically produced and cloned into pET28a(+) by using the NdeI and XhoI restriction sites, producing the lysis inhibitor from phage F379 with an N-terminal his-tag.

#### 2.2.6. Construction of pAZ06

A 988 bp fragment including *F379_024* and the downstream overlapping *lys (F379_025)* plus an additional TAA stop codon was synthetically produced and cloned into pET28a(+) by using the NdeI and XhoI restriction sites, producing the lysis inhibitor with an N-terminal his-tag and the endolysin from phage F379.

### 2.3. Protein Expression and Lytic Assays

The engineered plasmids were transformed into the expression strain, *E. coli* BL21-CodonPlus (DE3)-RIL (Agilent Technologies, Santa Clara, CA, USA) (Table S1), and transformants were selected in the presence of kanamycin (100 μg/mL) and chloramphenicol (50 μg/mL). Transformants were verified by PCR and sequencing using the universal T7 promoter and terminator primers. Single colonies of *E. coli* carrying the engineered plasmids were grown overnight in Difco™ LB Broth, Lennox (LB) (Becton, Dickinson and Company, Sparks, MD, USA) in the presence of kanamycin (100 μg/mL) and chloramphenicol (50 μg/mL). Cells were subcultured in a deep well plate containing 200 μL of LB broth and were grown to the mid-logarithmic growth phase (OD_600_ = 0.4). Expression of proteins was induced by using a final concentration of 1 mM isopropyl-beta-D-thiogalactopyranoside (IPTG, Thermo Fisher Scientific, Waltham, MA, USA) and turbidities were measured spectrophotometrically at 600 nm every 10 min for two hours by a Microplate Elisa Reader Gen5 (Biotek, Winooski, VT, USA). To investigate whether the endolysins use the Sec-pathway for their translocation to periplasm, lytic assays were conducted in the presence of sodium azide (NaN_3_). In this case, expression of proteins was induced in *E. coli* by 1 mM IPTG in the presence of 5 mM NaN_3_, and cell turbidity was analyzed spectrophotometrically, as mentioned above.

## 3. Results

### 3.1. In Silico Identification of Lytic Genes in Fletchervirus Phages

**Figure 1:**
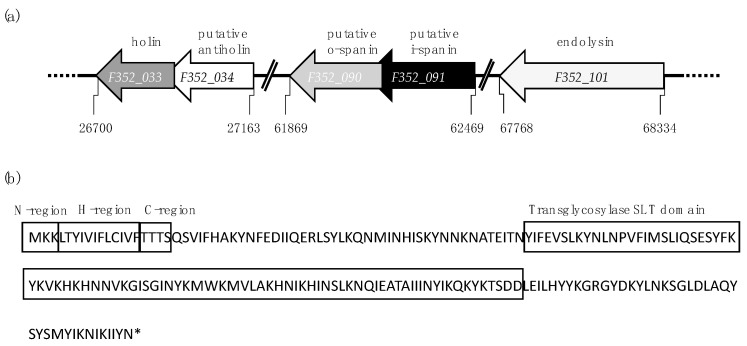
Illustration of lytic genes predicted in *Fletchervirus* phage F352. (**a**) Relative positions of predicted lytic genes in the genome of phage F352. (**b**) Secondary structure of phage F352 endolysin. The endolysin is predicted to possess a signal peptide (with N, H and C regions) that is located distantly from the catalytic soluble transglycosylase (SLT) domain.

Phages infecting Gram-negative hosts commonly code for three functional classes of lytic proteins: holins, endolysins and spanins [1]. Additionally, antiholins inhibiting the function of holin have been found in this group of phages [9]. To predict the lytic genes encoded by the *Fletchervirus* phages, we performed a detailed in silico analysis of the genome sequence of phage F352 selected as a representative of this genus (Table 1 and Table S2, Figure 1a).

To identify a putative endolysin, the F352 genome was analyzed for domains with lytic activity (excluding the virion-associated peptidoglycan hydrolase). The endolysin gene *lys (F352_101)* could be identified based on its soluble lytic transglycosylase (SLT) catalytic domain (PF01464) (Table 1). The endolysin possesses a clear N-terminal signal sequence with all three regions (N, H and C) located distantly from the catalytic domain (Figure 1b and Figure S1) and thus is predicted to be a typical SP endolysin [14].

Most phages of Gram-negative hosts have two-component spanins, comprised of an integral inner membrane i-spanin, and an outer membrane lipoprotein o-spanin, that together form a complex that spans the periplasm by C-terminal interactions [17]. Similarly, we predicted that phage F352 encodes such a two-component spanin system in an overlapping architecture, with i-spanin containing a TMD and o-spanin being an outer-membrane lipoprotein (Figure 1a).

Generally, holins display low homology to other holins and are often identified based on being integral membrane proteins encoded near or adjacent to other lytic genes. While the lytic genes of F352 are not organized into a cassette, the holin could be identified based on the presence of a well-defined LydA holin motif (IPR032126) (Table 1). The holin has class I topology (3 TMDs, N-out and C-in topology), as shown in Supplementary Table S3. Additionally, we identified an overlapping gene *F352_034* downstream of the holin. In phage P1, the stop codon of LydA holin overlaps with the start codon of LydB that functions as an antiholin [41]. Based on this overlapping architecture, we predict that *F352_034* may also function as an antiholin. Analysis of all the nine available sequenced *Fletchervirus* phages demonstrates that all the predicted lytic genes are conserved within this genus, with the exception of the spanins in phage Cp39 that display no homology at the DNA level with the predicted phage F352 spanins (Supplementary Table S4). However, these spanins do show more than 90% homology at the protein level (Supplementary Table S5). In summary, our in silico analysis indicates that *Fletchervirus* phages encode an SP-endolysin with a lytic transglycosylase domain, a holin and a putative antiholin, as well as a two-component spanin, none of which are organized in a lysis cassette.

### 3.2. In Silico Identification of Lytic Genes in Firehammervirus Phages

**Figure 2:**
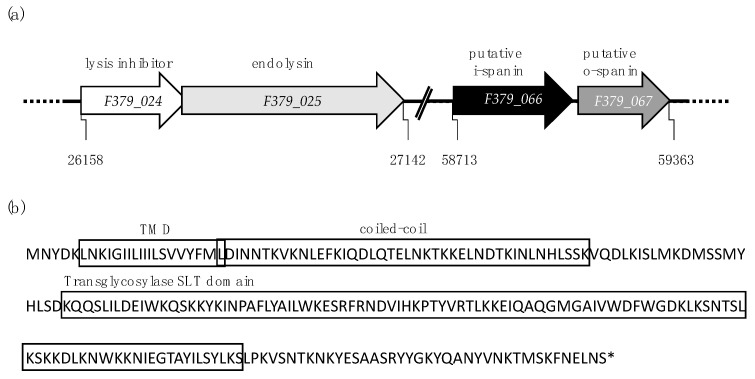
Illustration of lytic genes predicted in *Firehammervirus* phage F379. (**a**) Relative positions of predicted lytic genes in the genome of phage F379. (**b**) Secondary structure of phage F379 endolysin. The endolysin contains an N-terminal transmembrane domain linked to the catalytic soluble transglycosylase (SLT) domain by a sequence containing a coiled-coil motif. No known lysis mechanism has been described for an endolysin with this topology.

To identify the lytic genes in *Firehammervirus* phages, the same principles as above were applied, using phage F379 as a representative of this genus (Table 2 and Table S2, Figure 2a).

The endolysin was identified based on a soluble lytic transglycosylase (SLT) catalytic domain (PF01464) located at the C-terminus and it corresponds to the previously annotated endolysin in the *Firehammervirus* phage vB_CcoM-IBB_35 [32]. Here, we found that this endolysin has a novel architecture (Figure 2b and Figure S1). Unlike the *Fletchervirus* phages, there is no predicted N-terminal SP sequence. Rather, an N-terminal TMD and a coiled-coil motif (amino acids 24-65) are predicted. Unlike the TMDs of reported SAR endolysins, with weakly hydrophobic or polar residues, the TMD found in phage F379 endolysin consists of strongly hydrophobic amino acids. No known lysis mechanism has been reported for an endolysin with such structure. Gene *F379_024* is located upstream the predicted endolysin, with fourteen nucleotides overlapping the beginning of the *lys* gene. Furthermore, *F379_024* is conserved in all *Firehammervirus* (Figure 2a and Supplementary Table S4). While bioinformatic analysis provided no clues about its function, its conservation and close association with the endolysin suggests that it might have a role in lysis regulation.

Phage F379 also encodes a two-component spanin complex (Table 2 and Figure 2a). The spanins were identified based on their adjacent but non-overlapping gene architecture, with the predicted i-spanin containing a TMD and o-spanin being an outer-membrane lipoprotein. A search for small TMD-containing proteins revealed several holin candidates (*F379_006*, *F379_007*, *F379_008*, *F379_020*, *F379_196* and *F379_206*). However, no holin-associated domains or homology to confirmed holins were detected. Furthermore, analysis of the predicted holin of *Firehammervirus* phage IBB_35 and its homolog in phage F379 failed to detect the previously reported Phage_holin_3 family (PF05106.5) domain [32]. Thus, a putative holin candidate could not be predicted for *Firehammervirus* phages via in silico analysis. Analysis of all available sequenced *Firehammervirus* phages demonstrated that the predicted lytic genes are conserved within this genus. However, it was not possible to identify spanins in phage IBB_35, as this part of the genome, which is next to a region of repeated DNA sequences, is missing from the published contigs (Supplementary Table S4). In summary, our in silico analysis indicates that *Firehammervirus* phages encode a novel endolysin with a unique architecture, a lysis inhibitor overlapping the endolysin and a two-component spanin complex, while a holin candidate could not be identified.

### 3.3. Fletchervirus Phages Utilize a SP Endolysin System

**Figure 3:**
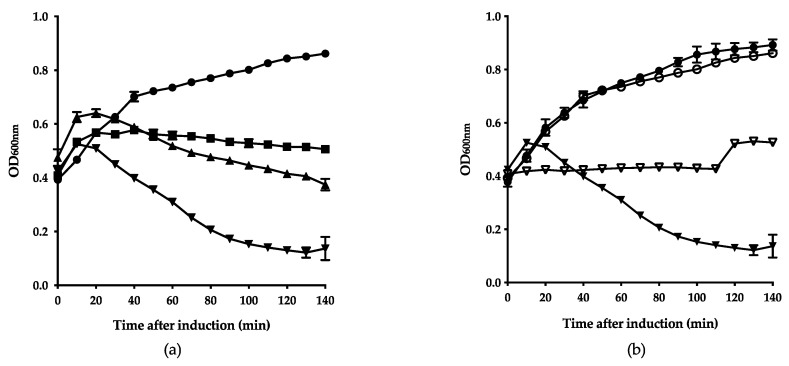
*Fletchervirus* phage F352 endolysin uses the Sec-pathway to access the periplasm, followed by activation by the holin. (**a**) Growth of *E. coli* cells expressing predicted lytic proteins. Optical density (OD_600_) of cells expressing endolysin (■); holin (▲) and co-expression of endolysin and holin (▼) was compared to *E. coli* cells carrying empty vector (●). (**b**) Dependence of predicted endolysin on the Sec-pathway. To investigate the dependence of the endolysin on the Sec-system, cells expressing the endolysin and holin were cultured in the presence of sodium azide (NaN3) (▽) or absence (▼). The growth of cells carrying the empty vector with (○) and without (●) NaN_3_ served as negative controls.

To confirm the bioinformatic predictions for the holin and endolysin in *Fletchervirus* phages, the two lytic genes from F352 were expressed in *E. coli*, as no expression system is available for *Campylobacter*, and both species share a common peptidoglycan chemotype (A1γ) [42]. To assess the impact of the predicted lytic genes on the bacterial growth, the optical density of culture growth was measured after induction of protein expression (Figure 3a).

Expression of endolysin slowed cell growth compared to the cells carrying the empty vector, putatively due to the metabolic burden of the overexpression interfering with the bacterial growth. Expression of holin led to growth inhibition, which is consistent with previous findings that show that holins inhibit cell growth when expressed alone [5,6]. Co-expression of the endolysin and holin led to lysis of the cells, confirming that the endolysin requires the holin for targeting the peptidoglycan layer. To determine whether the endolysin uses the predicted SP to target the Sec-pathway in order to reach the periplasm, lytic assays were performed with a SecA motor protein inhibitor, sodium azide (Figure 3b). Co-expression of endolysin and holin in the presence of the sodium azide inhibited cell lysis, confirming that indeed the endolysin uses the Sec-system for translocation. Overall, these data confirm the in silico predictions and demonstrate that the endolysin utilizes a SP to access the periplasm and is activated by the holin, leading to peptidoglycan degradation and cell lysis.

### 3.4. Firehammervirus Phages Display a Novel Lysis Mechanism

**Figure 4:**
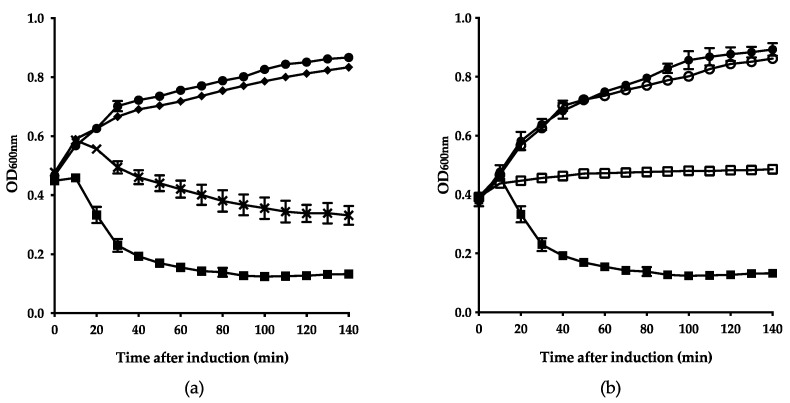
*Firehammervirus* phage F379 endolysin relies on the Sec-pathway for translocation but causes cell lysis independent of a holin. (**a**) Growth of *E. coli* cells expressing predicted lytic proteins. Optical density (OD_600_) of cells expressing endolysin (■); lysis inhibitor (◆) and co-expression of lysis inhibitor and endolysin (**×**) was compared to *E. coli* cells carrying empty vector (●). (**b**) Dependence of the predicted endolysin on the Sec-pathway. To investigate the dependence of the endolysin on the Sec-system, cells expressing the endolysin were cultured in the presence of sodium azide (NaN_3_) (☐) or absence (■). The growth of cells carrying the empty vector with (○) and without (●) NaN_3_ served as negative controls.

To confirm the role of the predicted lytic proteins in *Firehammervirus* phages, using F379 as a representative, lytic assays were conducted as above. Induction of the endolysin alone led to cell lysis within the initial 30 min (Figure 4a), indicating that the overexpression of the endolysin may be toxic for cells.

Our in silico analysis identified a small gene (*F379_024)* with no known motifs located upstream and overlapping with the endolysin (Table 2 and Figure 2a). In some phages, genes located adjacent or overlapping with endolysins have a regulatory function in the cell lysis [13]. To investigate whether *F379_024* has a role in lysis regulation, we expressed *F379_024* alone or co-expressed in combination with the endolysin. Expression of *F379_024* alone did not influence the growth compared to the negative control (induced *E. coli* carrying the empty vector). However, when the endolysin was co-expressed with *F379_024*, cell lysis was delayed and reduced compared to expression of the endolysin alone (Figure 4a). These results suggest that *F379_024* works as a lysis inhibitor, delaying and preventing maximum endolytic activity of the endolysin. Dependence of the endolysin on the Sec-system was investigated using a lytic assay in the presence of sodium azide as with the endolysin from phage F352 (Figure 4b). Interestingly, expression of the endolysin together with sodium azide led to growth inhibition instead of cell lysis, demonstrating that the endolysin is dependent on the Sec-pathway for translocation. In summary, the F379 endolysin uses the Sec-pathway to access the peptidoglycan in the periplasm, while a novel lysis inhibitor appears to regulate cell lysis by an unknown mechanism.

## 4. Discussion

The genomes of *Campylobacter* phages offer a reservoir of unexplored and novel genes. Lytic genes are of vital importance for phage propagation, ensuring the release of newly produced phages from the infected cells. However, lytic genes in *Campylobacter* phages have not been thoroughly studied because of the weak protein homology to other phages and the lack of genomic organization [23]. As a result, lytic genes are often missed during annotation, and the exact mechanism of lysis regulation remains unclear. Here, we predicted the lytic genes in *Campylobacter* phages F352 and F379 that served as representatives of *Fletchervirus* and *Firehammervirus* genera, respectively. We experimentally confirmed the function of the predicted lytic genes and we further performed a homology search in all publicly available *Campylobacter* phage genomes. Our in silico analysis confirmed that lytic genes are conserved within each genus, and our data demonstrated that the two genera utilize different types of lysis mechanisms.

*Fletchervirus* phages encode a typical SP transglycosylase and a class I holin to lyse the cells. Endolysins with SPs target the Sec-pathway in order to reach the periplasm. Here, we confirmed that phage F352 encodes a SP transglycosylase that requires the Sec-system for translocation to the periplasm and a holin for activation as described in the *Oenococcocus oeni* phage fOg44 endolysin (Lys44) [10,11]. Previous analysis on *Fletchervirus* phage vB_CjeM_los1 identified a homolog of F352 *lys*, Los1_127, and was predicted to function as a signal-arrest-release (SAR) endolysin [30]. However, the presence of a signal sequence with all three regions (N, H and C) situated distantly from the catalytic domain and strongly hydrophobic residues in the H-region are not characteristics of SAR endolysins, rather, these are characteristics of SP-endolysins [14]. We further identified that phage F352 *hol* is homologous to Los1_58, which was not previously predicted as a holin, rather Los1_53 was incorrectly annotated as a pinholin solely due to its small size.

In contrast to *Fletchervirus*, we showed that the endolysin in *Firehammervirus* phages was sufficient to lyse the *E. coli* cells after induction. Lysis of *E. coli* cells caused by expression of SAR endolysins alone has been reported both in coliphage P1 and *Pseudomonas aeruginosa* phage φKMV after 60–90 min of induction. Such cell lysis has been attributed to spontaneous release of the tethered endolysins from the inner membrane to the periplasm. Further co-expression of the endolysins with the holins accelerated bacterial cell lysis within 15 min of induction [15,16]. In *Firehammervirus* phages, cell lysis started rapidly after 20 min of endolysin expression alone. However, several putative holin candidates were predicted that could control cell lysis during phage infection of the native *Campylobacter* host. Furthermore, no SAR domain was predicted in the *Firehammervirus* endolysin. In contrast to SAR endolysins, the N-terminal part of the F379 endolysin has a transmembrane domain with strong hydrophobic residues, which are typical for SP endolysins. However, no SP was identified, indicating that this endolysin has a novel composition with the first 40 residues not matching any available endolysin sequences. Interestingly, we did find that cell lysis caused by the endolysin was inhibited in the presence of sodium azide, supporting that the endolysin uses the Sec-pathway for translocation, which is a conserved feature for both SP and SAR endolysins. However, the exact mode of how the *Firehammervirus* endolysin targets the Sec-system remains unclear. In addition, we found that the transmembrane domain of the endolysin is connected to the catalytic domain by a seventy-two-amino-acids-long sequence containing a domain with a coiled-coil motif (forty-two amino acids). This motif is a characteristic of spanins rather than endolysins and is normally associated with protein–protein interactions [43,44]. Several endolysins require dimerization with themselves or with other gene products to be activated during phage infection of Gram-positive bacteria [45,46,47,48]. *Clostridia* endolysin CTP1L, for instance, is suggested to be activated after side-by-side dimers formation, triggering the cleavage of the C-terminal domain and the release of the activated catalytic domain of the endolysin [48]. A similar interaction may occur in *Firehammervirus* endolysin regulating its function. Thus, all these characteristics support the notion that *Firehammervirus* endolysin indeed has a novel architecture with a putative novel lysis function not previously described for phages infecting Gram-negative bacteria.

Interestingly, we demonstrated that *F379_024* partially overlapping the endolysin in *Firehammervirus* phages inhibited the lytic activity of the endolysin after co-expression, suggesting that it works as a lysis inhibitor. Phages use holins to control lysis by allowing the access of endolysin to the periplasm or by activating the periplasm-faced endolysins [6]. Here, it appears that *Firehammervirus* phages have evolved a novel mechanism to control the cell lysis by utilizing a lysis inhibitor to suppress the already-active endolysins that gain access to the periplasm by the Sec-pathway. A lysis regulator located near to an endolysin has also been described for *Mycobacterium smegmatis* phage Ms6 [13]. In this case, a holin was also not required for cell lysis; however, here the lysis regulator acted as an activator of the endolysin by targeting the endolysins to the Sec-pathway. It remains unclear whether the *Firehammervirus* lysis inhibitor interacts directly with the endolysin either at the DNA or protein level, and the exact mode of action is yet to be clarified. However, the coiled-coil motif in the endolysin suggests that protein–protein interaction may occur, either leading to the multimerization of the endolysin or/and is required for the interaction with the lysis inhibitor.

## Figures and Tables

**Table 1:** Lytic genes predicted in *Fletchervirus* phage F352.

Size (aa ^1^)	Gene	Function	Characteristics
93	*hol (F352_033)*	holin	Class I (3 TMDs ^2^), LydA-like holin (IPR032126)
61	*F352_034*	putative antiholin	C-terminal TMD, N-in C-out topology
97	*F352_090*	putative o-spanin	Outer membrane lipoprotein (PS51257) motif with SP ^3^ sequence (Phobius), overlapped with Rz
112	*F352_091*	putative i-spanin	N-terminal TMD with coiled-coil motif
188	*lys (F352_101)*	endolysin	Transglycosylase SLT endolysin (PF01464) with SP

^1^ Amino acid; ^2^ Transmembrane domains, ^3^ Signal peptide.

**Table 2:** Lytic genes predicted in *Firehammervirus* phage F379.

Size (aa ^1^)	Gene	Function	Characteristics
106	*F379_024*	lysis inhibitor	Homologs are found in all *Firehammervirus* located upstream and partially overlapping with endolysin
224	*lys (F379_025)*	endolysin	Transglycosylase SLT endolysin (PF01464) with 1 TMD ^2^, N-out C-in topology and coiled-coil motif
120	*F379_066*	putative i-spanin	N-terminal TMD with coiled-coil motif
92	*F379_067*	putative o-spanin	Outer membrane lipoprotein (PS51257) motif with SP ^3^ sequence (Phobius), separated by Rz

^1^ Amino acid; ^2^ Transmembrane domains, ^3^ Signal peptide.

## Supplementary Materials

The following are available online at https://www.mdpi.com/1999-4915/12/11/1247/s1.

## References

[1] Young R. (2013). Phage lysis: Do we have the hole story yet?. Curr. Opin. Microbiol..

[2] Young R. (2014). Phage lysis: Three steps, three choices, one outcome. J. Microbiol..

[3] Fernández-Ruiz I., Coutinho F.H., Rodriguez-Valera F. (2018). Thousands of Novel Endolysins Discovered in Uncultured Phage Genomes. Front. Microbiol..

[4] Oliveira H., Melo L.D., Santos S.B., Nobrega F.L., Ferreira E.C., Cerca N., Azeredo J., Kluskens L.D. (2013). Molecular aspects and comparative genomics of bacteriophage endolysins. J. Virol..

[5] Wang I.-N. (2006). Lysis Timing and Bacteriophage Fitness. Genetics.

[6] Ing-Nang W., David L.S., Young R. (2000). Holins: The Protein Clocks of Bacteriophage Infections. Annu. Rev. Microbiol..

[7] Young R. (1992). Bacteriophage lysis: Mechanism and regulation. Microbiol. Rev..

[8] Tran T.A.T., Struck D.K., Young R. (2005). Periplasmic Domains Define Holin-Antiholin Interactions in T4 Lysis Inhibition. J. Bacteriol..

[9] Gründling A., Smith D.L., Bläsi U., Young R. (2000). Dimerization between the Holin and Holin Inhibitor of Phage λ. J. Bacteriol..

[10] São-José C., Parreira R., Vieira G., Santos M.A. (2000). The N-Terminal Region of the Oenococcus oeni Bacteriophage fOg44 Lysin Behaves as a Bona Fide Signal Peptide in *Escherichia coli* and as a cis-Inhibitory Element, Preventing Lytic Activity on Oenococcal Cells. J. Bacteriol..

[11] Nascimento J.G., Guerreiro-Pereira M.C., Costa S.F., São-José C., Santos M.A. (2008). Nisin-Triggered Activity of Lys44, the Secreted Endolysin from Oenococcus oeni Phage fOg44. J. Bacteriol..

[12] Xu M., Arulandu A., Struck D.K., Swanson S., Sacchettini J.C., Young R. (2005). Disulfide Isomerization After Membrane Release of Its SAR Domain Activates P1 Lysozyme. Science.

[13] Catalão M.J., Gil F., Moniz-Pereira J., Pimentel M. (2010). The mycobacteriophage Ms6 encodes a chaperone-like protein involved in the endolysin delivery to the peptidoglycan. Mol. Microbiol..

[14] von Heijne G. (1990). The signal peptide. J. Membr. Biol..

[15] Briers Y., Peeters L.M., Volckaert G., Lavigne R. (2011). The lysis cassette of bacteriophage varphiKMV encodes a signal-arrest-release endolysin and a pinholin. Bacteriophage.

[16] Kuty G.F., Xu M., Struck D.K., Summer E.J., Young R. (2010). Regulation of a phage endolysin by disulfide caging. J. Bacteriol..

[17] Summer E.J., Berry J., Tran T.A.T., Niu L., Struck D.K., Young R. (2007). Rz/Rz1 Lysis Gene Equivalents in Phages of Gram-negative Hosts. J. Mol. Biol..

[18] Krupovic M., Cvirkaite-Krupovic V., Bamford D.H. (2008). Identification and functional analysis of the Rz/Rz1-like accessory lysis genes in the membrane-containing bacteriophage PRD1. Mol. Microbiol..

[19] Berry J., Rajaure M., Pang T., Young R. (2012). The spanin complex is essential for lambda lysis. J. Bacteriol..

[20] Bieńkowska-Szewczyk K., Taylor A. (1980). Murein transglycosylase from phage λ lysate purification and properties. BBA.

[21] Rajaure M., Berry J., Kongari R., Cahill J., Young R. (2015). Membrane fusion during phage lysis. Proc. Natl. Acad. Sci. USA.

[22] Ackermann H.-W. (2009). Phage classification and characterization. Bacteriophages.

[23] Hammerl J.A., Jäckel C., Reetz J., Beck S., Alter T., Lurz R., Barretto C., Brüssow H., Hertwig S. (2011). *Campylobacter jejuni* group III phage CP81 contains many T4-like genes without belonging to the T4-type phage group: Implications for the evolution of T4 phages. J. Virol..

[24] Sørensen M.C.H., Gencay Y.E., Birk T., Baldvinsson S.B., Jäckel C., Hammerl J.A., Vegge C.S., Neve H., Brøndsted L. (2015). Primary isolation strain determines both phage type and receptors recognised by *Campylobacter jejuni* bacteriophages. PLoS ONE.

[25] Javed M.A., Ackermann H.-W., Azeredo J., Carvalho C.M., Connerton I., Evoy S., Hammerl J.A., Hertwig S., Lavigne R., Singh A. (2014). A suggested classification for two groups of *Campylobacter* myoviruses. Arch. Virol..

[26] Kropinski A.M., Arutyunov D., Foss M., Cunningham A., Ding W., Singh A., Pavlov A.R., Henry M., Evoy S., Kelly J. (2011). Genome and proteome of *Campylobacter jejuni* bacteriophage NCTC 12673. Appl. Environ. Microbiol..

[27] Timms A.R., Al Khandari S., Wilson R., Rowsell J., Connerton I.F. (2011). *Campylobacter* Phage CPX, Complete Genome.

[28] Janež N., Peterka M., Accetto T. (2016). Complete Genome Sequences of Group III *Campylobacter* Bacteriophages PC5 and PC14. Genome Announc..

[29] Loc Carrillo C., Atterbury R.J., El-Shibiny A., Connerton P.L., Dillon E., Scott A., Connerton I.F. (2005). Bacteriophage Therapy to Reduce *Campylobacter jejuni* Colonization of Broiler Chickens. Appl. Environ. Microbiol..

[30] O’Sullivan L., Lucid A., Neve H., Franz C., Bolton D., McAuliffe O., Paul Ross R., Coffey A. (2018). Comparative genomics of Cp8viruses with special reference to *Campylobacter* phage vB_CjeM_los1, isolated from a slaughterhouse in Ireland. Arch. Virol..

[31] Hammerl J.A., Jäckel C., Reetz J., Hertwig S. (2012). The complete genome sequence of bacteriophage CP21 reveals modular shuffling in *Campylobacter* group II phages. J. Virol..

[32] Carvalho C.M., Kropinski A.M., Lingohr E.J., Santos S.B., King J., Azeredo J. (2012). The genome and proteome of a *Campylobacter coli* bacteriophage vB_CcoM-IBB_35 reveal unusual features. Virol. J..

[33] Timms A.R., Cambray-Young J., Scott A.E., Petty N.K., Connerton P.L., Clarke L., Seeger K., Quail M., Cummings N., Maskell D.J. (2010). Evidence for a lineage of virulent bacteriophages that target *Campylobacter*. BMC Genom..

[34] Finn R.D., Attwood T.K., Babbitt P.C., Bateman A., Bork P., Bridge A.J., Chang H.-Y., Dosztányi Z., El-Gebali S., Fraser M. (2017). InterPro in 2017—Beyond protein family and domain annotations. Nucleic Acids Res..

[35] Krogh A., Larsson B., von Heijne G., Sonnhammer E.L. (2001). Predicting transmembrane protein topology with a hidden Markov model: Application to complete genomes. J. Mol. Biol..

[36] Tsirigos K.D., Peters C., Shu N., Käll L., Elofsson A. (2015). The TOPCONS web server for consensus prediction of membrane protein topology and signal peptides. Nucleic Acids Res..

[37] NCBI Resource Coordinators (2015). Database resources of the National Center for Biotechnology Information. Nucleic Acids Res..

[38] Finn R.D., Clements J., Arndt W., Miller B.L., Wheeler T.J., Schreiber F., Bateman A., Eddy S.R. (2015). HMMER web server: 2015 update. Nucleic Acids Res..

[39] Nielsen H. (2017). Predicting Secretory Proteins with SignalP. Methods Mol. Biol. (Clifton, N.J.).

[40] Söding J., Biegert A., Lupas A.N. (2005). The HHpred interactive server for protein homology detection and structure prediction. Nucleic Acids Res..

[41] Schmidt C., Velleman M., Arber W. (1996). Three functions of bacteriophage P1 involved in cell lysis. J. Bacteriol..

[42] Schleifer K.H., Kandler O. (1972). Peptidoglycan types of bacterial cell walls and their taxonomic implications. Bacteriol. Rev..

[43] Cahill J., Rajaure M., Holt A., Moreland R., O’Leary C., Kulkarni A., Sloan J., Young R. (2017). Suppressor Analysis of the Fusogenic Lambda Spanins. J. Virol..

[44] Burkhard P., Stetefeld J., Strelkov S.V. (2001). Coiled coils: A highly versatile protein folding motif. Trends Cell Biol..

[45] Nelson D., Schuch R., Chahales P., Zhu S., Fischetti V.A. (2006). PlyC: A multimeric bacteriophage lysin. Proc. Natl. Acad. Sci. USA.

[46] McGowan S., Buckle A.M., Mitchell M.S., Hoopes J.T., Gallagher D.T., Heselpoth R.D., Shen Y., Reboul C.F., Law R.H.P., Fischetti V.A. (2012). X-ray crystal structure of the streptococcal specific phage lysin PlyC. Proc. Natl. Acad. Sci. USA.

[47] Proenca D., Velours C., Leandro C., Garcia M., Pimentel M., Sao-Jose C. (2015). A two-component, multimeric endolysin encoded by a single gene. Mol. Microbiol..

[48] Dunne M., Mertens H.D.T., Garefalaki V., Jeffries C.M., Thompson A., Lemke E.A., Svergun D.I., Mayer M.J., Narbad A., Meijers R. (2014). The CD27L and CTP1L Endolysins Targeting Clostridia Contain a Built-in Trigger and Release Factor. PLoS Pathog..

