# Supplementary material for: Two Distinct Modes of Lysis Regulation in Campylobacter Fletchervirus and Firehammervirus Phages
Source: Viruses. 2020 Oct 31;12(11):1247. doi: 10.3390/v12111247 (PMC7692668; doi:10.3390/v12111247)
Supplement: Supplementary file 1 [file viruses-12-01247-s001.pdf]

viruses

Supplementary Materials

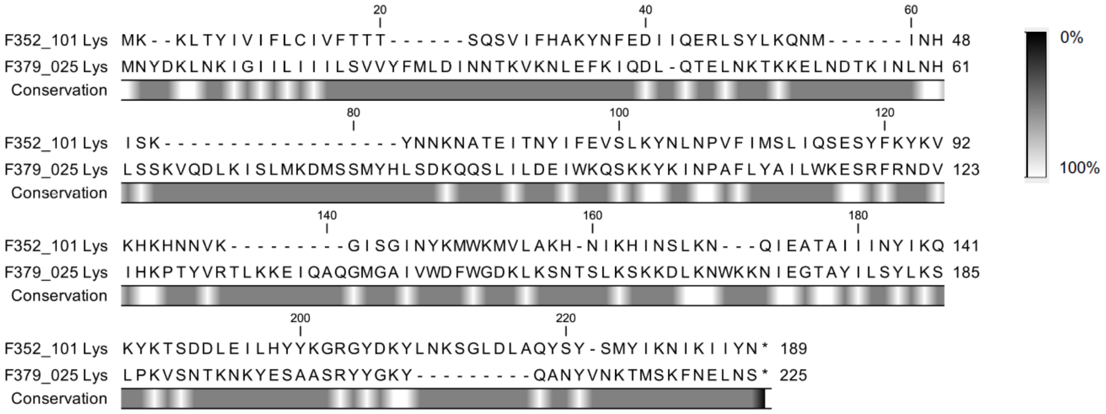

Figure S1. Protein sequence alignment of the endolysins from phage F352 and F379. The alignment was performed using QIAGEN CLC main workbench version 20.0.4 using standard settings.

Table S1. Bacterial strains, phages and plasmids.

***E. coli* strains**	**Description**		**Reference**
**BL21-CodonPlus (DE3)-RIL Competent Cells**	**Codon optimized protein expression strain, Cam^r (50 µg/ml)**		**Agilent Technologies**
AZ01	BL21-CodonPlus (DE3)-RIL+pAZ01, *lys (F352_101)* protein expression strain, Kan^r (100 µg/ml), Cam^r (50 µg/ml)		This study
AZ02	BL21-CodonPlus (DE3)-RIL+pAZ02, *hol (F352_033)* protein expression strain, Kan^r (100 µg/ml), Cam^r (50 µg/ml)		This study
AZ03	BL21-CodonPlus (DE3)-RIL+pAZ03, *lys (F352_101)* and *hol (F352_033)* protein co-expression strain, Kan^r (100 µg/ml), Cam^r (50 µg/ml)		This study
AZ04	BL21-CodonPlus (DE3)-RIL+pAZ04, *lys (F379_025)* protein expression strain, Kan^r (100 µg/ml), Cam^r (50 µg/ml)		This study
AZ05	BL21-CodonPlus (DE3)-RIL+pAZ05, *F379_024* protein expression strain, Kan^r (100 µg/ml), Cam^r (50 µg/ml)		This study
AZ06	BL21-CodonPlus (DE3)-RIL+pAZ06, *F379_024* and *lys (F379_025)* protein co-expression strain, Kan^r (100 µg/ml), Cam^r (50 µg/ml)		This study
**Phages**	**Description**	**Genbank accession no.**	**Reference**
**F352**	Genome size: 131.638 bp<br>Family: *Myoviridae*; Subfamily: *Eucampyvirinae*;<br>Genus: *Fletchervirus*	MT863717	[1]
**F379**	Genome size: 183.102 bp<br>Family: *Myoviridae*; Subfamily: *Eucampyvirinae*;<br>Genus: *Firehammervirus*	MT932329	[1]
**Plasmid**	**Characteristics**		**Reference**
pET28a+	Protein expression vector:<br>N-His, N-Thrombin, C-His , Kan^r (100 µg/ml)		Novagen
pAZ01	*lys (F352_101)* expression plasmid<br>pET28a+:: *lys (F352_101)* from phage F352, Kan^r (100 µg/ml)		This study
pAZ02	*hol (F352_033)* expression plasmid<br>pET28a+:: *hol (F352_033)* from phage F352, Kan^r (100 µg/ml)		This study
pAZ03	*lys (F352_101)* and *hol (F352_033)* co-expression plasmid<br>pET28a+:: *lys (F352_101)* and *hol (F352_033)* from phage F352, Kan^r (100 µg/ml)		This study
pAZ04	*lys (F379_025)* expression plasmid<br>pET28a+:: *lys (F379_025)* from phage F379, Kan^r (100 µg/ml)		This study
pAZ05	*F379_024* expression plasmid<br>pET28a+:: *F379_024* from phage F379, Kan^r (100 µg/ml)		This study
pAZ06	*F379_024* and *lys (F379_025) co*-expression plasmid<br>pET28a+:: *F379_024* and *lys (F379_025)* from phage F379, Kan^r (100 µg/ml)		This study

Table S2. Lytic genes predicted in *Fletchervirus* phage F352 and *Firehammervirus* phage F379.

Phage	*gene*	Position	Strand	Size (aa[1])	Protein Mw (kDa)	Function	Characteristics
**F352**							
	lys (F352_101)	67768-68334	-	188	22,4	endolysin	Lysozyme-like domain superfamily (IPR023346) Transglycosylase SLT domain (PF01464) Transglycosylase SLT domain 1 (IPR008258) (G3DSA:1.10.530.10) (SSF53955) Signal peptide region (1-19) Phobius, N-Region (aa 1-3), H-region (aa 4-15), C-region (aa 16-19)
	F352_091	62131-62469	-	112	13,4	putative Rz IM spanin (i-spanin)	N-terminal TMD coiled-coil motif
	F352_090	61869-62162	-	97	11,5	putative Rz1 OM spanin (o-spanin)	Outer membrane lipoprotein, PROKAR_LIPOPROTEIN (PS51257) motif and signal peptide sequence (Phobius)
	F352_034	26978-27163	-	61	7,8	putative antiholin	C-terminal TMD[2], N-in C-out topology.
	hol (F352_033)	26700-26981	-	93	10,4	holin	LydA-like holin (IPR032126) Phage_holin_3_3 (PF16083) 3 TMDs, N-out C-in topology (Class I holin)
**F379**							
	F379_024	26158-26478	+	106	12,6	lysis inhibitor	Homologs are found in all *Firehammervirus* located upstream and partially overlapping with endolysin
	lys (F379_025)	26468-27142	+	224	26,3	endolysin	Lysozyme-like domain superfamily (IPR023346) Transglycosylase SLT domain (PF01464) Transglycosylase SLT domain 1 (IPR008258) (G3DSA:1.10.530.10) (SSF53955) 1 TMD, N-out C-in topology coiled-coil motif (aa 24-65)

F379_066	58713-59075	+	120	13,5	putative Rz IM spanin (i-spanin)	N-terminal TMD coiled-coil
F379_067	59085-59363	+	92	10,9	putative Rz1 OM spanin (o-spanin)	Outer membrane lipoprotein, PROKAR_LIPOPROTEIN (PS51257) motif and signal peptide sequence (Phobius)

[1] Amini acid

[2] Transmembrane domain

Table S3. Phage F352 holin predicted Transmembrane (TM)-helix positions.

	**TMI**	**TM2**	**TM3**
TMHMM	5-24	37-59	60-93
TOPCONS	4-24	38-58	64-84
OCTOPUS	4-24	38-58	64-84
Philius	7-23	37-57	67-84
PolyPhobius	6-23	37-57	63-81
SCAMPI	3-23	38-58	61-81
SPOCTOPUS	4-24	38-58	64-84

Table S4. Lytic gene homologs found in all publicly available *Campylobacter* phage genomes at DNA level.

Phages	Blast Coordinates	Identities	E-value
***Fletchervirus* phages[1]**			
**holin**			
CP81	77246-77527	276/282 (98%)	4e-134
vB_CjeM_Los1	50889-50608	271/282 (96%)	8e-126
PC14	133282-133001	271/282 (96%)	8e-126
NCTC12673	98290-98009	271/282 (96%)	8e-126
CP39	119460- 119179	269/282 (95%)	2e-122
PC5	48229- 48510	269/282 (95%)	2e-122
CP8	51462-51181	276/282 (98%)	4e-134
CPX	51228- 50947	276/282 (98%)	4e-134
CP30A	98930- 98649	267/282 (95%)	4e-119
**antiholin**			
CP81	77064-77249	183/186 (98%)	5e-86
vB_CjeM_Los1	51071-50886	185/186 (99%)	2e-89
PC14	133464-133279	185/186 (99%)	2e-89
NCTC12673	98472-98287	185/186 (99%)	2e-89
CP39	119642-119457	178/186 (96%)	1e-77
PC5	48047-48232	178/186 (96%)	1e-77
CP8	51644-51459	183/186 (98%)	5e-86
CPX	51410-51225	183/186 (98%)	5e-86
CP30A	99112-98927	184/186 (99%)	1e-87
**o-spanin**			
CP81	38860-39153	290/294 (99%)	4e-144
vB_CjeM_Los1	87090- 86797	289/294 (98%)	2e-142
PC14	34642- 34349	290/294 (99%)	4e-144
NCTC12673	134685-134392	290/294 (99%)	4e-144
CP39	-	-	-
PC5	15021-15314	289/294 (98%)	2e-142
CP8	89470-89177	288/294 (98%)	8e-141
CPX	89234-88941	288/294 (98%)	8e-141
CP30A	3143-2850	287/294 (98%)	4e-139
**i-spanin**			
CP81	38553-38891	333/339 (98%)	9e-166
vB_CjeM_Los1	87397-87059	330/339 (97%)	1e-160
PC14	34949-34611	333/339 (98%)	9e-166
NCTC12673	134992-134654	333/339 (98%)	9e-166
CP39	-	-	-
PC5	14714-15052	335/339 (99%)	4e-169
CP8	89777-89439	335/339 (99%)	4e-169
CPX	89541-89203	335/339 (99%)	4e-169
CP30A	3450- 3112	333/339 (98%)	9e-166
**endolysin**			
CP81	33260-33826	554/567 (98%)	0.0
vB_CjeM_Los1	93255-92689	552/567 (97%)	0.0

PC14	41440-40874	552/567 (97%)	0.0
NCTC12673	6442-5876	552/567 (97%)	0.0
CP39	28491-27925	551/567 (97%)	0.0
PC5	8853- 9419	550/567 (97%)	0.0
CP8	95660-95094	550/567 (97%)	0.0
CPX	95424-94858	550/567 (97%)	0.0
CP30A	9315-8747	549/569 (96%)	0.0
	***Firehammervirus* phages**[a]		
**lysis inhibitor**			
CP21	172813-172493	316/321 (98%)	2e-157
CP220	106817-107134	311/318 (98%)	2e-152
CPt10	107232-107552	317/321 (99%)	4e-159
vB_CcoM-IBB_35	18666-18346	307/321 (96%)	2e-142
**endolysin**			
CP21	172503-171828	673/676 (99%)	0.0
CP220	107127-107801	651/675 (96%)	0.0
CPt10	107542-108216	649/675 (96%)	0.0
vB_CcoM-IBB_35	18356-17682	637/675 (94%)	0.0
**i-spanin**			
CP21	180703-181064	358/363 (99%)	4e-180
CP220	98700- 99062	344/363 (95%)	2e-157
CPt10	99030-99392	350/363 (96%)	2e-167
vB_CcoM-IBB_35	-	-	-
**o-spanin**			
CP21	181074-181352	279/279 (100%)	2e-142
CP220	99072-99350	253/279 (91%)	4e-99
CPt10	99402- 99680	254/279 (91%)	8e-101
vB_CcoM-IBB_35	-	-	-

[1]Accession numbers of both *Fletchervirus* and *Firehammervirus* phages: CP81 (FR823450.1); vB_CjeM_Los1 (KX879627.1); PC14 (KX236333.1); NCTC12673 (GU296433.1); CP39 (MH107028.1); PC5 (KX229736.1); CP8 (KF148616.1); CPX (JN132397.1); CP30A(JX569801.1); CP21 (HE815464.1); CP220 (FN667788.1); vB_CcoM-IBB_35 (HM246724.1).

Table S5. Homologs of phage F352 spanins found in phage Cp39 at the protein level.

	Identities	Positives	Gaps	E-value
**i-spanin**	77/112(69%)	92/112(82%)	0/112(0%)	4e-52
**o-spanin**	75/97(77%)	91/97(93%)	0/97(0%)	4e-57

Reference

1. Sørensen, M.C.H.; Gencay, Y.E.; Birk, T.; Baldvinsson, S.B.; Jäckel, C.; Hammerl, J.A.; Vegge, C.S.; Neve, H.; Brøndsted, L. Primary isolation strain determines both phage type and receptors recognised by *Campylobacter jejuni* bacteriophages. *PloS one* **2015**, *10*, e0116287.
